# 

**Published:** 1857-12

**Authors:** 


### CONTENTS.

ORIGINAL COMMUNICATIONS— PAGE.

Address, Introductory to the Annual Course of Lectures in Rush Medical College. By N. S. Davis, M.D. 535
Ununited Fractures. By Charles Brackett, M.D. 548
Cases of Cancer treated at the College Clinic by Prof. Brainard. Reported by Edwin Powell, M.D. 552
Report of Case Involving Loss of Corresponding Portions of both Hemispheres of the Brain. By L. N. Dimmick, M.D. 557
Chloroform in Paroxysmal Typhoid Fever. By F. W. White, M.D. 559
Translations for the Journal. By Dr. Byford.
Death Caused by Hemorrhage from the Vaginal Mucous Membrane... 564
Abortive Treatment of Extrauterine Fœtation by Means of Electricity. By Dr. Burci of Pisa 565
Treatment of Phthisis Pulmonalis 566
Congenital Idiopathic Cerebro-Spinal Meningitis. By M. Stoltz 567

PROCEEDINGS OF MEDICAL SOCIETIES—

Annual Meeting of the Esculapian Society 570
New Castle (Ind.) Medical Society 574

BOOK NOTICES—

The Medical Student's Vade Mecum. By G. Mendenhall, M.D 576
The Handbook of Practical Receipts for every day use. By T. F. Branston. 576
A Collection of Remarkable Cases of Surgery. By P. F. Eve. 577

EDITORIAL—

Close of the Volume 578
Rush Medical College Clinic 578

